# Determination of total and unbound docetaxel in plasma by ultrafiltration and UPLC-MS/MS: application to pharmacokinetic studies

**DOI:** 10.1038/s41598-017-15176-0

**Published:** 2017-11-03

**Authors:** Ming-Thau Sheu, Chen-Yuan Wu, Chia-Yu Su, Hsiu-O Ho

**Affiliations:** School of Pharmacy, College of Pharmacy, Taipei Medical University, 250 Wu-Hsing Street, Taipei, 11031 Taiwan

## Abstract

A sensitive and specific liquid chromatographic/tandem mass spectrometric (LC-MS/MS) method was developed and validated for quantifying total and unbound docetaxel drug concentrations in plasma. Calibration curves for unbound and total docetaxel were linear over the respective ranges of 0.108~10.8 and 0.54~216 ng/mL. The intra- and interday assay accuracy and precision did not exceed 15%. The methods were validated to show the standard range linearity, sensitivity, selectivity, accuracy, precision, and stability of docetaxel in the matrices tested. In addition, this method is fast and simple with a short run time of 4.5 min and a small plasma sample volume (500 µL). The validated method was successfully applied to a pharmacokinetic study of a docetaxel micelle formulation in rat plasma after intravenous administration at a dose of 10 mg/kg. Docetaxel micelles slowly released their drug payload, and protein-bound, unbound, and micellar drug pools existed simultaneously. These various forms in plasma pools were also measured in the study. We confirmed that most of the docetaxel in plasma was micelle-associated (96.52% at 24 h and 83.14% at 72 h) after micellar docetaxel administration, as a result of sequestration of the drug in long-circulating micelles.

## Introduction

Docetaxel is an antineoplastic agent that shows significant activity in ovarian, non-small-cell lung, breast, and head and neck cancers^1^. Several factors influence its efficacy and toxicity, and assessing an optimal dose is difficult due to inter-patient variability in pharmacokinetics (PKs)^2^. One of the factors inducing this variation is protein binding. Docetaxel is highly bound to proteins, with unbound fractions generally < 10% in plasma. Interestingly, it was recently reported that exposure to unbound docetaxel rather than the total drug level was more closely related than the total drug to drug-induced hematological toxicity^3^. The unbound, active fraction is more closely related to the pharmacological and/or toxic effect, and measuring the unbound fraction may therefore be more useful than the total plasma concentration. Numerous analytical methods with high-performance liquid chromatography (LC; HPLC) and LC-tandem mass spectrometry (LC-MS/MS) were developed to determine docetaxel in plasma samples^4–10^. These methods quantified the total amount of docetaxel in circulation. However, unbound concentrations of docetaxel may be better correlated with treatment outcomes (i.e., efficacy and toxicity) than is the total drug^11^. All methods use either equilibrium dialysis or ultrafiltration to separate the unbound drug from the protein-bound or encapsulated fractions. Further, adopting ultrafiltration for separating unbound drugs in PK studies has gained attention in recent years^12,13^ due to its rapidity, simplicity, and higher analytical throughput. Analytical methods using ultrafiltration followed by HPLC-MS/MS were reported for determining unbound docetaxel in biological samples^2,14^. Nevertheless, those methods have several shortcomings, such as a long single run time (10 min), a large volume of biological sample required (2 mL), poor sensitivity (lower limit of quantification (LOQ; LLOQ) of 10 or 0.4 ng/mL)^14–16^, and a restricted formulation of docetaxel^14^. Traditionally, docetaxel has been used in high doses every third week to treat cancers. Lately, there has been a trend towards giving lower weekly doses to improve the therapeutic index^8^. It is important to develop suitable HPLC methods to determine docetaxel concentrations in patients receiving low-dose therapy.

In addition, docetaxel is greatly limited in clinical use due to its high toxicity and poor water solubility. Ethanol and Tween 80 are often added as solubilizers, which can induce some unpredictable toxicities such as diarrhea, cumulative fluid retention, hypersensitivity, neurotoxicity, and neutropenia^17,18^. They also affect the binding of docetaxel in the plasma in a concentration-dependent manner, and influence the disposition of intravenously (iv) administered solubilized drugs. Therefore, there is a need to develop better drug delivery systems to overcome these shortcomings and allow determination of unbound drug concentrations to assess the PKs of docetaxel. Several drug carriers, including liposomes, microspheres, micelles, nanocapsules, etc., have been used to deliver docetaxel. An HPLC method was developed and applied to the PKs of docetaxel liposomes and a docetaxel injection after iv administration to rabbits. Calibration curves were linear over the range of 0.025~2.525 µg/mL for total docetaxel in plasma. The LOQ was 10.0 ng/mL. These results showed that liposome carriers led to a significant difference in tissue distributions and PK profiles compared to a traditional docetaxel injection^19^. However, several factors contributed to the complexity of the pharmacologic effects of the drug delivered by carriers after iv administration, including that the circulating drug was present in three different forms (e.g., protein-unbound, protein-bound, and carrier-associated). Because these pools differed in their PKs, efficacy, and safety, quantification of the total drug concentration alone was insufficient to characterize the biopharmaceutic properties of drugs loaded or encapsulated in carriers. In our previous studies, docetaxel-loaded mixed polymeric micelles showed improved efficacies in cancer therapy and reduced side effects^20^. It is necessary to establish a method for determining unbound docetaxel concentrations in plasma after administration by micelles, since we are unaware of any published literature describing this.

LC-MS/MS generally has better selectivity and sensitivity than HPLC. It is widely used because of its ability to accurately quantitate analytes with minimal sample clean-up and rapid separation. But LC-MS/MS methodologies occasionally encounter some problems caused by matrix effects^21,22^. Current US Food and Drug Administration (FDA) guidance documents now require that these effects be evaluated as a part of quantitative LC-MS/MS method development, validation, and routine use^23–25^. However, no analytical method describing the quantitation of unbound docetaxel in plasma has discussed matrix effects to any degree. The FDA documents further emphasize the urgent need for a more-thorough and -complete evaluation and solution to the problem of matrix effects. In the present study, the authors present the development and validation of a versatile and sensitive UPLC-MS/MS method to determine unbound and total docetaxel in plasma with the advantages of a short run time and small plasma sample volume. We also evaluated matrix effects (MEs) with the UPLC-MS/MS method. The method was used to carry out PK studies for docetaxel micelles in rats after iv administration. Furthermore, these data were used to estimate the docetaxel concentrations of plasma in various pools (e.g., unbound, protein-bound, and micellar-associated) after administration of docetaxel micelles.

## Results

### Chromatographic and MS conditions

**Figure 1:**
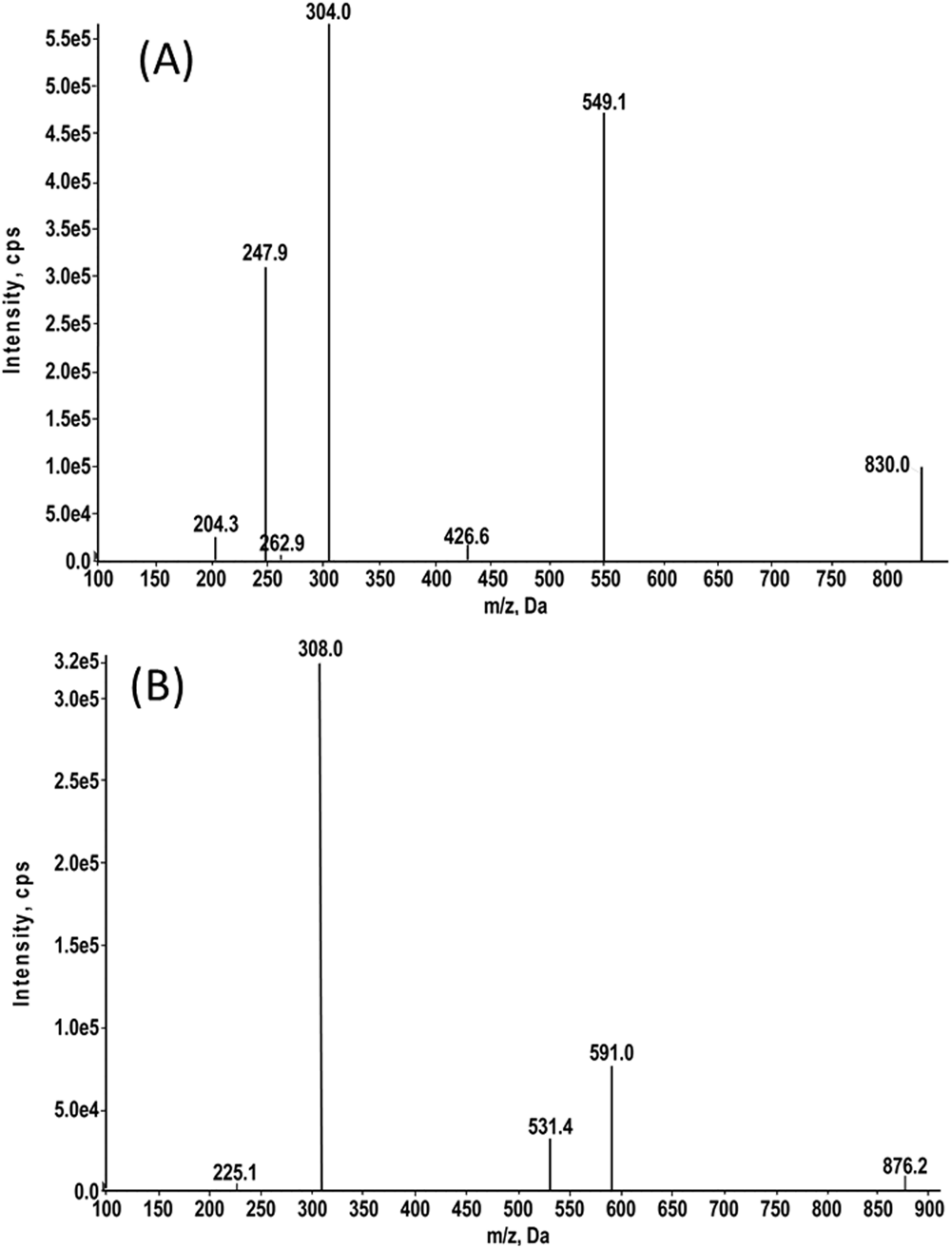
Full-scan product ion mass spectra of (**A**) docetaxel and (**B**) the internal standard (IS; paclitaxel).

To evaluate the sensitivity of both docetaxel and the IS, different solvent types and additives in various concentrations and ratios were tested. Better peak shapes and optimal sensitivity were obtained with the addition of 0.1% formic acid and acetonitrile as the organic solvents. Using the gradient elution mobile phase, all analytes were rapidly eluted within 6.0 min (Fig. 1S). Docetaxel and paclitaxel (the IS) were respectively eluted at 1.58 and 1.63 min.

Docetaxel and the IS could be ionized under either positive (ESI^+^) or negative (ESI^−^) ESI conditions^26^. In this study, ESI^+^ was used for detection due to its superior sensitivity over ESI^−^. Depending on different experimental conditions, docetaxel and the IS may tend to generate different adducts when ionized through an ESI source. Although there are reports that exploited protonated adduct MRM transitions with good sensitivity^27^, in our studies, spectra revealed that docetaxel formed Na^+^ adducts more easily than H^+^ adducts even in the presence of formic acid. Under ESI^+^ conditions respective precursor ions for docetaxel and the IS were [docetaxel + Na]^+^ at m/z 830.0 and [paclitaxel + Na]^+^ at m/z 876.2 as shown in Fig. 1. In the spectra, m/z 304.0 and 308.0 were the most sensitive product ions for docetaxel and the IS, respectively, and these were chosen as quantifier ions in subsequent detections. Product ions of m/z 549.1 and 591.0 produced the next highest intensities for docetaxel and the IS, respectively, and these were chosen as qualifier ions.

### Method validation

Chromatographic conditions had adequate specificity for docetaxel and the IS, while no endogenous interference peaks were observed at retention times of docetaxel (1.58 min) or the IS (1.63 min). Docetaxel and the IS were clearly separated from endogenous peaks originating from the blank matrix. Representative MRM chromatograms of blank plasma in mass transition showed that there was no background interference in the docetaxel or IS peak windows (data not shown).

**Figure 2:**
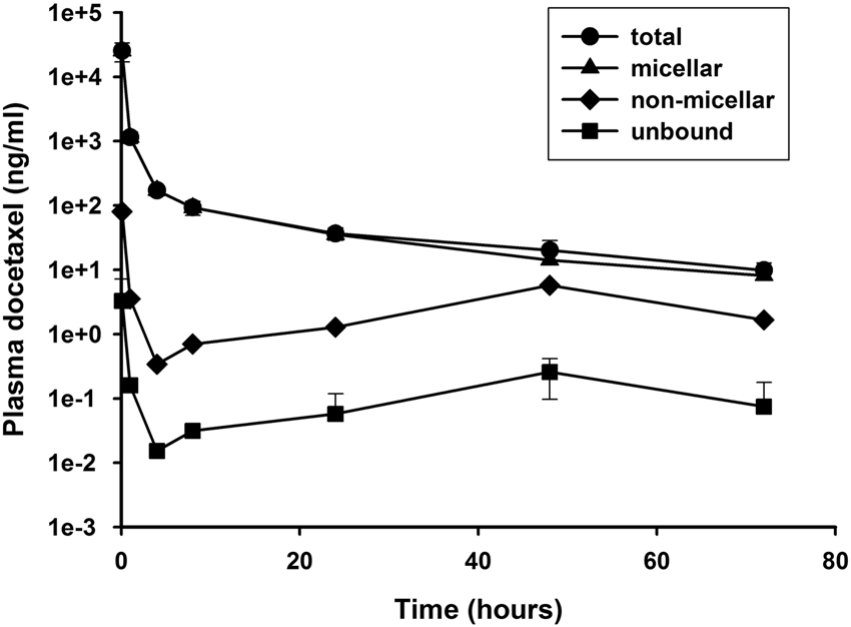
Docetaxel concentrations in various plasma pools after intravenous administration of 10 mg/kg of docetaxel micelles in SD rats.

Results of the attained intra- and inter-day precision and accuracy for docetaxel in ultrafiltered plasma and total plasma are summarized in Table 1. The intra- and inter-day precision levels of the analysis for docetaxel in ultrafiltered plasma were < 11.92%, with an accuracy of <  ± 12.72% for both (*n* = 6). And the intra- and inter-day precision levels of the analysis for docetaxel in total plasma were < 10.66%, with an accuracy of <  ± 12.70% for both (*n* = 6). These results showed that the analytical method is precise, accurate, and reproducible for detecting both docetaxel and the IS. The method exhibited excellent linearity over the ranges of 0.108~10.8 and 0.54~216 ng/ml in ultrafiltered and total plasma, respectively, with reproducible correlation coefficients of *r*^2^ > 0.99. The LLOQ concentration under the optimized condition was 0.108 ng/ml for docetaxel with a signal-to-noise (S/N) ratio of 19.6. The response was reproducible, and a stable baseline was maintained during the analysis with an S/N ratio of ≥ 10-fold the baseline. A chromatogram of docetaxel at the LLOQ concentration is presented in Fig. 2S.

**Table 1:** Intra-day and inter-day precision, and accuracy for docetaxel in ultrafiltered and total plasma.

Sample	Concentration (ng/mL)	Intra-day			Inter-day		
		Mean ± SD	CV (%)	RE (%)	Mean ± SD	CV (%)	RE (%)
Ultra-filtered plasma	0.108	0.122 ± 0.01	7.61	12.72	0.115 ± 0.01	11.92	6.63
	0.27	0.269 ± 0.02	6.77	−0.48	0.257 ± 0.02	7.88	−4.85
	0.54	0.476 ± 0.04	9.21	−11.91	0.507 ± 0.04	7.06	−6.13
	1.08	1.07 ± 0.05	5.05	−0.55	1.08 ± 0.07	6.07	0.29
	5.40	5.37 ± 0.12	2.33	−0.59	5.33 ± 0.13	2.41	−1.25
	10.8	10.89 ± 0.33	3.06	0.83	10.91 ± 0.40	3.64	0.98
Total plasma	0.54	0.60 ± 0.05	7.88	10.47	0.61 ± 0.05	8.74	12.70
	1.08	1.13 ± 0.09	8.09	5.04	1.13 ± 0.05	4.29	4.47
	5.4	4.84 ± 0.52	10.66	−10.32	4.80 ± 0.25	5.12	−11.10
	54	48.91 ± 4.52	9.24	−9.42	50.47 ± 3.52	6.98	−6.54
	108	111.73 ± 3.98	3.56	3.46	108.01 ± 4.78	4.42	0.01
	216	217.80 ± 6.88	3.16	0.83	220.06 ± 6.98	3.17	1.88

SD, standard deviation; CV, coefficient of variation; RE, relative error.

It is important that the carry-over effect and dilution integrity applied to measure drug concentrations in biological matrices be validated for bioanalytical methods. Results showed that carry-over was avoidable, and it did not affect the accuracy or precision of the bioanalytical methods. In addition, results of dilution integrity showed that the accuracy and precision were within set criteria, i.e., within ± 15% (Table 1). Dilution of samples did not affect the accuracy or precision.

### Assessment of ME/extraction efficiency

The purpose of sample preparation was to remove interferences and extract the analytes from the plasma samples. An economical, clean, and simple SPE method was developed to achieve requirements of clinical PKs in this study. As stated in the 2001 FDA guidelines, “In the case of LC-MS/MS-based procedures, appropriate steps should be taken to ensure the lack of matrix effects throughout the application of the method”^23^. Matuszewski *et al*.^25^ developed a method to quantitatively evaluate MEs in the analytical process. The analytical quality of the entire experimental process (i.e., process efficiency, PE) is related to the presence of interference during sample preparation and assay (e.g., ion transmission, ionization, etc.). Interference might also affect sample preparation, and lead to a decrease in the extraction yield (EY) compared to standards. The extraction recovery (ER) in the presence of the matrix should therefore be determined. Thus, a complete investigation of MEs in the studies required the evaluation of four ratios (ME, PE, ER, and EY), and these results are listed in Table 2. The ME (i.e., potential ion enhancement or suppression) of docetaxel, calculated as the area ratio with and without matrix ions present, was between 103.5 and 87.7 for ultrafiltered plasma, and between 111.5 and 99.4 for total plasma. These results showed that impurities, degradation products, or co-eluting matrix components did not affect the ionization of docetaxel. Elimination of MEs is critical to generate reliable bioanalytical and PK data. In the range of concentrations studied, the PEs for docetaxel in ultrafiltered and total plasma were > 90.0% and > 87.6%, respectively. As shown in Table 2, the ER and EY of docetaxel were about 89.4%~104.8% for ultrafiltered plasma and 88.1%~109.0% for total plasma, and were within acceptable ranges. Therefore, the extraction efficiency of docetaxel in ultrafiltered and total plasma was constant, precise, and reproducible.

**Table 2:** The matrix effect and extract efficiency of docetaxel in ultrafiltered and total plasma.

Parameter	Ultrafiltered plasma Concentration (ng/mL)			Total plasma Concentration (ng/mL)		
	0.27	1.08	10.8	2.16	54	216
Process efficiency (%) (PE)	100.8	90.0	91.6	106.4	104.7	87.6
Matrix effect (%) (ME)	103.5	87.7	102.5	105.6	111.5	99.4
Extraction recovery (%) (ER)	97.4	102.6	89.4	96.8	93.9	88.1
Extraction yield (%) (EY)	99.9	98.1	104.8	102.0	109.0	96.0

### Correlation between unbound and total docetaxel concentrations *in vitro*

Rat ultrafiltered and unfiltered plasma samples were spiked with docetaxel over a range of concentrations, concentrations of unbound (C_UF_) and total (C_TOT_) docetaxel were measured, and these results are listed in Table 3. Equation 1 was obtained using a nonlinear, least-squares parameter estimation method to fit these data:

1 $${{\rm{C}}}_{{\rm{UF}}}=35.03\,{\rm{x}}\,{{\rm{C}}}_{{\rm{TOT}}}/776.87+{{\rm{C}}}_{{\rm{TOT}}{\rm{.}}}$$

**Table 3:** Concentrations (ng/mL) of total (C_TOT_) and unbound (C_UF_) docetaxel in plasma (n = 7).

Spiked concentration	Measured concentration	
C_TOT_	C_TOT_	C_UF_
2.16	2.07 ± 0.89	0.16 ± 0.08
5.40	5.38 ± 0.57	0.29 ± 0.10
10.80	11.44 ± 2.19	0.46 ± 0.12
21.60	20.65 ± 2.97	0.99 ± 0.45
54.00	55.62 ± 3.08	2.42 ± 0.91
81.00	83.38 ± 10.2	3.31 ± 2.10
108.00	107.8 ± 3.70	4.24 ± 1.66
180.00	176.9 ± 15.6	6.52 ± 2.78

### PK studies

**Figure 3:**
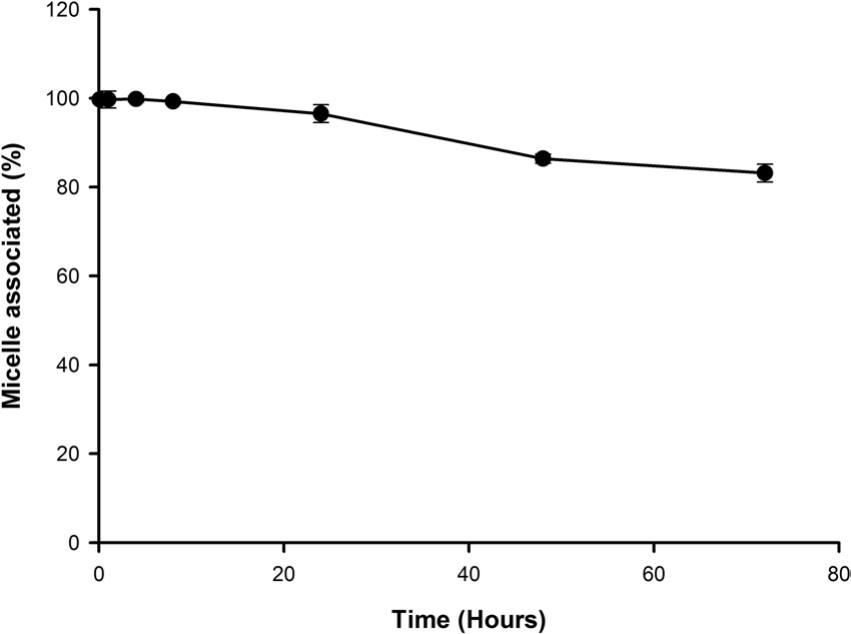
Percentage of docetaxel in plasma that was micelle-associated after intravenous administration of 10 mg/kg of docetaxel micelles in SD rats.

The validated UPLC-MS/MS method was applied to PK studies of the iv administration of Tynen and docetaxel micelles to SD rats. Profiles of the mean plasma docetaxel concentration versus time are illustrated in Fig. 3S. Figure 3S shows that the drug concentration in plasma rapidly decreased during the first 1.0 h, which is consistent with Zhao’s studies.^ref?^ Docetaxel plasma concentrations obtained at 0.083 h after iv administration were 25,365.00 ± 8342.03 ng/mL for micelles and 7519.00 ± 1955.05 ng/mL for Tynen. The docetaxel concentration of micelles in plasma showed an insignificant difference with Tynen at each time point except for the first 0.083 h. And the drug concentration in plasma was detectable at up to 72 h using the analytical method established in this study. The main PK parameters are presented in Table 4. The respective half-lives of Tynen and micelles were 26.47 ± 22.54 and 24.56 ± 8.19 h. The AUC of micelles was about 2.0 times greater than that of Tynen, and the clearance decreased after iv administration of micelles. The results indicated that micelles could postpone elimination and lead to a longer blood circulating effect of the drug in rats.

**Table 4:** Pharmacokinetic parameters of total docetaxel in rats receiving intravenous Tyne or docetaxel micelle at a dose of 10 mg/kg.

Formulation	T_1/2_	C_max_	AUC_0~72_	AUC_0~inf_	V	Cl	MRT
	(h)	(ng/ml)	(h × ng/ml)	(h × ng/ml)	(L/kg)	(L/h/kg)	(h)
Tynen	26.47 ± 22.54	10,426 ± 3275	6748 ± 1603	7293 ± 1594	49.95 ± 36.82	1.42 ± 0.31	6.37 ± 1.72
Micelles	24.56 ± 8.19	39,085 ± 14,356	14,055 ± 3241	14,425 ± 3310	25.20 ± 7.87	0.72 ± 0.17	4.54 ± 0.16

Data are presented as the mean ± standard deviation (*n* = 3). T_1/2_, half-life of the drug; C_max_, maximum plasma drug concentration; AUC_0~72_, area under the receiver operating curve at 0~72 h; AUC_0~inf_, area under the receiver operating curve at 0 to infinity; V, volume of the distribution; Cl, clearance; MRT, mean residence time.

### Concentration profiles of docetaxel micelles in various plasma pools

Profiles of docetaxel concentrations in various plasma pools after iv administration of 10 mg/kg docetaxel micelles in SD rats versus time are shown in Fig. 2. Concentrations of mean unbound drug fell from 3.91 ± 3.29 ng/mL at 0.083 h after the end of the infusion, to < 0.2 ng/mL by 24 h, and they remained between 0.1 and 0.2 ng/mL for the rest of the 72-h study. Concentrations of unbound docetaxel did not exceed 10 ng/mL at any time point in any subject administrated micellar docetaxel. Concentrations of non-micellar (protein-bound plus unbound) docetaxel were estimated from the measured unbound concentrations and percent binding data from the *in vitro* binding study; however, the non-micellar pool of docetaxel present in the plasma after micellar docetaxel administration was almost entirely protein-bound. Concentrations of non-micellar docetaxel after administration of 10 mg/kg micellar docetaxel remained at < 100 ng/mL in all subjects, with concentrations falling from 80.39 ng/mL immediately after dosing to < 5 ng/mL after 72 h (Fig. 2). Micellar docetaxel was then estimated by the difference between total and non-micellar docetaxel. Figure 3 shows that the percentage of docetaxel in plasma that was micelle-associated (%) slowly fell over the course of the study, from 99.68% at the end of the infusion to 83.14% at the end of the 72-h study. Overall, these results evidence that micellar docetaxel is a stable, long-circulating micellar delivery system that sequesters its docetaxel payload in the plasma, slowly releasing it over time as well as targeting the drug to cancer cells via the uptake of intact micelles.

## Discussion

It is recognized that the analytical technique of a drug is of upmost importance in dealing with clinical PKs, either in the general field of drug monitoring or more specifically studying anticancer drugs. This is particularly apparent when examining drugs delivered by carriers in pharmacological oncology. Since unbound, protein-bound, and carrier-associated forms may exist simultaneously within the body after administration of an anticancer drug delivered by a carrier., measurement of total docetaxel concentrations alone in plasma after administration by docetaxel micelle is insufficient to fully characterize the PK, safety, and efficacy profiles. However, the unbound form of docetaxel is more closely related to the efficacy and toxicity. In this study, we developed a sensitive and specific LC-MS/MS method to quantify unbound and total docetaxel drug concentrations in plasma. Using the optimized gradient mobile phase, docetaxel and the IS were respectively eluted at 1.58 and 1.63 min. The method performed well with respect to method validation, including both precision and accuracy with < 13% in ultrafiltered and total plasma, and was linear for unbound and total docetaxel over the respective ranges of 0.108~10.8 and 0.54~216 ng/mL, with an LLOQ of 0.108 ng/ml for docetaxel. In addition, evaluating matrix effects on the results of quantitative unbound and total docetaxel concentrations in plasma is important and required not only by validation standards but more importantly, is critical to generate reliable bioanalytical and pharmacokinetic data. The results were satisfactory and were not affected by the matrix effect on quantification in these studies. Overall the bioanalytical methods described above were validated to show the standard range linearity, sensitivity, selectivity, accuracy, precision, and stability of docetaxel in the matrices tested. In addition, advantages of this method also included the rapid, simple, and selective quantification of unbound and total docetaxel especially at low docetaxel concentrations. The validated method was applied to a PK study of a docetaxel micelle formulation in rat plasma after intravenous administration at a dose of 10 mg/kg. This study demonstrated that docetaxel was simultaneously present in different forms, including protein-bound, unbound, and micellar-associated after docetaxel micelle administration. The non-micellar (protein-bound plus unbound) and unbound docetaxel pools had much lower concentrations than the micellar pool after docetaxel micelle administration, consistent with the slow release of docetaxel and targeting the drug to cancer cells via uptake of intact micelles. At present, our studies provide a preclinical safety evaluation of intravenously administrated docetaxel micelles with determination of total and unbound docetaxel concentrations in plasma. In the future, we hope that the validated bioanalytical method can be further performed by including clinical trials in patients administered docetaxel micelles.

## Conclusions

A specific and sensitive analytical method with ultrafiltration and UPLC-MS/MS for determining both unbound and total concentrations of docetaxel in plasma was developed. The method can monitor plasma concentrations of as low as 0.108 ng/mL, and it is fast and simple with a short run time of 4.5 min and a small plasma sample volume (500 µL). In the study, this method was successfully validated and applied to determine both total and unbound docetaxel plasma concentrations from rats receiving a docetaxel micelle formulation at a dose of 10 mg/kg. Furthermore, concentrations of non-micellar and micellar docetaxel were also determined, and results confirmed that docetaxel micelles were highly associated (>80%) in plasma as a result of sequestration of the drug in long-circulating micelles.

## Methods

### Calibration and method validation

Two separate stock solutions of docetaxel from independent weighings were prepared in methanol at a concentration of 1080 µg/mL. One set of stock solutions was used to prepare calibration standards in methanol, while the other set was used to prepare calibration curves of total and unbound docetaxel in plasma. The IS stock solution was 1180 µg/mL paclitaxel in methanol. A calibration curve of docetaxel in methanol was prepared at concentrations of 21.6, 54, 810, 1080, and 1800 ng/mL by further dilution of the stock solution with methanol. Calibration curves for the total and unbound forms of docetaxel in plasma were prepared using the same procedures. First, appropriate amounts of the docetaxel stock solution and IS were spiked into drug-free rat plasma (for total docetaxel) or control ultrafiltered plasma (for the unbound form of docetaxel), then 1% formic acid was added and mixed well. Second, these mixtures were deproteinized using SPE as follows. With the help of a vacuum, the mixture was then passed through preconditioned (with 1 mL of methanol and 2 mL water) Waters Oasis HLB solid-phase cartridges (Milford, MA, USA). The cartridges were then washed with 1 mL of 1% formic acid and 1 mL of 50% methanol (containing 0.1% formic acid). Then, docetaxel and the IS were eluted with methanol (containing 0.1% formic acid). After an SPE, the organic layer was evaporated, and the residue was redissolved in 120 µL of 90% MeOH (containing 0.1% formic acid); it was then ready for the UPLC-MS/MS analysis. Finally, calibration curves of the total and unbound forms of docetaxel in plasma were prepared at concentrations of 0.54, 1.08, 5.4, 54, 108, and 216 ng/mL and 0.108, 0.27, 0.54, 1.08, 5.4, and 10.8 ng/mL, respectively. All stock and working solutions were stored at −20 °C and brought to ambient temperature before the analysis.

Responses of plasma samples were determined using the ratio of the peak area of each analyte to that of the IS. The ratio of the peak area (y) was plotted against analyte concentrations (x), and calibration curves were constructed in the form of y = A + Bx. Plasma calibration curves were prepared and assayed in triplicate on three different days to demonstrate the linearity of this method. Recovery was assessed by comparing the means of sample replicates to the aforementioned expected concentrations. The coefficient of variation (CV) and relative error (RE) of the mean were used to validate the intra- and inter-day precision and accuracy by determining standard samples of docetaxel in plasma. The LLOQ, defined as the lowest concentration measurable on the calibration curve that could be measured with acceptable accuracy and precision, was determined in six replicates on three consecutive days.

During validation, the carry-over effect was assessed by injecting blank samples after a high concentration sample or calibration standard at the upper limit of quantification (ULOQ). Dilution integrity was demonstrated by spiking the matrix (ultrafiltered plasma and total plasma, respectively) with docetaxel concentration above the ULOQ and diluting this sample with blank matrix (at least five determinations per dilution factor).

### ME characterization and extraction efficiency

To evaluate the absolute ME, blank and ultrafiltered plasma samples were extracted and then spiked with docetaxel at three quality control (QC) concentrations. Corresponding peak areas of docetaxel in post-extraction spiked plasma (B) were then compared to those of neat standards in the mobile phase (A) at equivalent concentrations. The ME was calculated by the ratio (B/A × 100). The process efficiency (PE) of docetaxel was estimated by comparing the corresponding peak areas of docetaxel in pre-extraction spiked plasma (C) with those obtained from the neat standard prepared in the mobile-phase solvent (A) at equivalent concentrations. PE was determined by the ratio (C/A × 100). The plasma extraction recovery (ER) of docetaxel was obtained by comparing the peak area of samples spiked before extraction (C, pre-extraction spiked plasma) to those of plasma samples spiked after extraction (B, post-extraction spiked plasma): ER = C/B × 100 according to Matuszewski *et al*.^24,25^. The extraction yield (EY) was determined with the corresponding peak areas of docetaxel in the neat extraction standard (D) to those of the neat standard in the mobile phase (A) at equivalent concentrations. The ratio (D/A × 100) was defined as the EY.

### Establishment of the correlation between unbound and total docetaxel concentrations *in vitro*

Unfiltered and ultrafiltered plasma samples were assayed by LC-MS/MS to obtain the total (C_TOT_) and unbound (C_UF_) concentrations of docetaxel and establish a correlation of total and unbound docetaxel concentrations in plasma. Sample processing procedures are depicted in Fig. 4S. Unfiltered and ultrafiltered plasma samples were both analyzed using the same analytical conditions described above.

For plasma samples spiked with docetaxel, the unbound docetaxel concentration (C_UF_, ultrafilterable) in ultrafiltered plasma plotted versus the total docetaxel concentration in plasma (C_TOT_, total) was fit to the C_UF_ = (A × C_TOT_)/(B + C_TOT_) equation using a nonlinear, least-squares parameter estimation method (Sigmaplot Software, Salt Lake City, UT, USA). The calculated parameters, A and B, were used to obtain docetaxel concentrations in various plasma pools in docetaxel micelle-treated rats.

### Applications to PK studies

SD rats (male, 7 weeks old) were purchased from BioLASCO Taiwan (Taipei, Taiwan) and used for PK studies. They were housed under a 12-h light/dark cycle with free access to food and water. All animal experimental methods were conducted in accordance with guidelines and regulations established by Taipei Medical University (Taipei, Taiwan). The experimental protocols in the animal study (approval no. LAC-2013-0137) were approved by the Laboratory Animal Center of Taipei Medical University.

Formulations were administered through iv routes with each drug administered at 10 mg/kg for Tynen and docetaxel micelles. Whole-blood samples were collected at predetermined time points. Plasma samples were obtained by centrifuging blood samples at 3000 rpm and 4 °C for 10 min; the plasma was then transferred to a new tube and stored at −80 °C until the analysis. These plasma samples were divided into two groups. One was for measuring total docetaxel concentrations, and the other was for unbound docetaxel concentrations in plasma. Total docetaxel plasma concentrations in rats administrated Tynen or docetaxel micelles were analyzed as follows. Plasma (100 µL) was mixed with 500 ng/mL paclitaxel (10 µL) as the IS and 1% formic acid and vigorously mixed. The sample was further deproteinized using SPE as described above. After the SPE, the organic layer was evaporated, and the residue was redissolved in 120 µL of 90% MeOH (containing 0.1% formic acid) and was then ready for injection into the UPLC-MS/MS instrument. Procedures for analyzing unbound docetaxel plasma concentrations in rat administered Tynen or docetaxel micelles were as follows. Plasma (500 µL) was placed inside a collection cup with a 30-kDa cutoff and centrifuged at 4 °C by rotating at 5270 *g* for 45 min in a model Allegra X-12R centrifuge (Beckman Coulter). Then, 150 µL of the supernatant was transferred to a microcentrifuge tube and mixed well with 59 ng/mL paclitaxel (15 µL) as the IS and 1% formic acid (150 µL). The next steps included deproteination and redissolvation based on the analytical procedures of total docetaxel plasma concentrations described above.

The related PK parameters, including the half-life of the drug (T_1/2_), maximum concentration of drug in blood plasma (C_max_), area under the receiver operating curve at 0~72 h (AUC_0~72_), AUC at 0 to infinity (AUC_0~inf_), volume distribution (V), clearance (Cl), and mean residence time (MRT), were analyzed using noncompartmental methods provided by WinNonlin software (vers. 6.3.0.395, Pharsight^®^, Princeton, NJ, USA).

### Estimation of docetaxel in various plasma pools

When conventional docetaxel is applied to rats, the majority of docetaxel is bound to plasma proteins, and the remaining unbound fraction is available to the target site. Therefore, the total concentration (C_TOT_) of docetaxel is the sum of that bound to proteins (C_PT_) and the unbound fraction (ultrafilterable, C_UF_) (shown in Equation 2). However, when micelles are used to deliver docetaxel to SD rats, non-micellar (the sum of protein-bound and unbound, C_non-micellar_) and micellar (C_micellar_) drug pools may exist simultaneously within the body. Since these pools differ in their PK, safety, and efficacy profiles, measuring every pool’s concentration is important. Equation 3 shows the relationship of docetaxel concentrations after administration of docetaxel micelles in plasma pools.

2 $${{\rm{C}}}_{{\rm{TOT}}}={{\rm{C}}}_{{\rm{PT}}}+{{\rm{C}}}_{{\rm{UF}}}({\rm{for}}\,{\rm{conventional}}\,{\rm{drug}})$$

3 $${{\rm{C}}}_{{\rm{TOT}}}={{\rm{C}}}_{{\rm{non}}-{\rm{micellar}}}+{{\rm{C}}}_{{\rm{micellar}}}={{\rm{C}}}_{{\rm{PT}}}+{{\rm{C}}}_{{\rm{UF}}}+{{\rm{C}}}_{{\rm{micellar}}}({\rm{for}}\,{\rm{micellar}}\,{\rm{carriers}})$$

Total (C_TOT_) and unbound (ultrafilterable, C_UF_) docetaxel concentrations were measured from the analyzed total and ultrafiltered rat plasma, respectively. The concentration of non-micellar docetaxel (C_non-micellar_) was defined as the sum of protein-bound (C_PT_) and unbound (C_UF_) drug in each micellar docetaxel-treated SD rat’s plasma. The above calculated parameters, A and B, were used to estimate concentrations of non-micellar docetaxel in plasma (C_non-micellar_) after administration of docetaxel micelles. The micellar docetaxel concentration was then calculated as the difference between the total and non-micellar docetaxel concentrations. The micelle-associated percentage (%) was defined as the percentage of docetaxel concentration in micelles after iv administration of docetaxel micelles and calculated using the following formula:

$${\rm{Micelle}} \mbox{-} {\rm{associated}}\,( \% )=100\times ({{\rm{C}}}_{{\rm{TOT}}}-\,{{\rm{C}}}_{{\rm{non}}-{\rm{micellar}}})/{{\rm{C}}}_{{\rm{TOT}}}.$$

## Electronic supplementary material

Supplementary information
