# Publisher Correction: Determination of total and unbound docetaxel in plasma by ultrafiltration and UPLC-MS/MS: application to pharmacokinetic studies

**DOI:** 10.1038/s41598-018-19644-z

**Published:** 2018-01-18

**Authors:** Ming-Thau Sheu, Chen-Yuan Wu, Chia-Yu Su, Hsiu-O Ho

**Affiliations:** School of Pharmacy, College of Pharmacy, Taipei Medical University, 250 Wu-Hsing Street, Taipei, 11031 Taiwan

Correction to: *Scientific Reports* 10.1038/s41598-017-15176-0, published online 03 November 2017

The original HTML version of this Article contained a typographical error in the publication date ‘03 November 2017’ which was incorrectly given as ‘06 November 2017’. This has now been corrected in the HTML version of the Article.

