# Supplementary material for: Determination of total and unbound docetaxel in plasma by ultrafiltration and UPLC-MS/MS: application to pharmacokinetic studies
Source: Sci Rep. 2017 Nov 3;7:14609. doi: 10.1038/s41598-017-15176-0 (PMC5668284; doi:10.1038/s41598-017-15176-0)
Supplement: Supplementary file 1 — Supplementary information [file 41598_2017_15176_MOESM1_ESM.doc]

**Supplementary Information:**

**Title of manuscript:**

Determination of total and unbound docetaxel in plasma by ultrafiltration and UPLC-MS/MS: application to pharmacokinetic studies

**Authors:**

Ming-Thau Sheu, Chen-Yuan Wu, Chia-Yu Su, Hsiu-O Ho*

Correspondence and requests for materials should be addressed to Hsiu-O Ho

(email: hsiuoho@tmu.edu.tw)

**Supplementary information includes:**

Supplemental information includes supplemental materials, apparatus and UPLC-MS/MS conditions; one table and four figures can be found with this article online.

**Supplemental Materials**

Docetaxel was purchased by Scino Pharm (Tainan, Taiwan). Paclitaxel (the internal standard, IS), acetonitrile, formic acid, and methanol were from Sigma-Aldrich (St. Louis, MO, USA). Amicon ultra-0.5-mL centrifugal filters, with an approximate 30-kDa molecular weight cutoff, were obtained from Millipore (St. Louis, MO, USA) and placed in a collection cup of an ultrafiltration device for the ultrafiltration procedure. Control ultrafiltered plasma was prepared in-house from control EDTA rat plasma by ultrafiltration with the filters. Approximately 0.5 mL of EDTA rat plasma was placed inside the collection cup and centrifuged at 4 °C by rotating at 3270 *g* for 20 min in a model Allegra X-12R centrifuge (Beckman Coulter, Indianapolis, IN, USA). All reagents used for high-performance liquid chromatographic (LC; HPLC) analyses, including acetonitrile, methanol, and formic acid, were HPLC grade, and other reagents were analytical grade. LC/mass spectrometry (MS)-grade water was purchased from Scharlau (Barcelona, Spain). Docetaxel micelles were obtained from and characterized by Prof. Ming-Thau Sheu of Taipei Medical University (Taipei, Taiwan).

**Apparatus and ultrapure LC tandem MS (UPLC-MS/MS) conditions**

Chromatography was performed using an ACQUITY UPLC system equipped with a Kinetex C18 column (50 mm × 2.1 mm i.d., 1.7 um particle size; Phenomenex, Torrance, CA, USA). The gradient elution mobile phase was composed of (A) a 0.1% formic acid-water mixture and (B) 0.1% formic acid-acetonitrile delivered at a flow rate of 0.3 ml/min. The gradient was controlled as follows: 70% A → 10% A at 0~2.00 min; 10% A at 2.00~4.00 min; 10% A → 70% A at 4.00~4.01 min; and 70% A at 4.01~6.00 min. The column temperature was maintained at 40 °C, and the autosampler was set to 4 °C. A 2.0-µl aliquot was injected into the UPLC-MS/MS system, and the total analytical run time was 4.5 min.

The MS analysis was performed on a QTRAP 6500 mass spectrometer (AB Sciex, Concord, Canada) equipped with an electrospray ionization (ESI) source in the positive ionization mode. The ion spray voltage (ISV) was set to 5500 V. The optimal turbo-gas temperature (TEM) was set to 400 °C, and the curtain gas (CUR) at 40.0 psi, ion source gas 1 (nebulizer) at 50.0 psi, and turbo ion source gas 2 (heater) at 60.0 psi were optimized. Signals of analytes and the IS were monitored through multiple reaction monitoring (MRM). A complete overview of the MS/MS transitions, declustering potential (DP), collision energy (CE) voltages, and collision cell exit potential (CXP) are compiled in Table 1S. Quadrupoles Q1 and Q3 were maintained at unit resolution with a dwell time of 100 ms per channel. Data acquisition and peak integration were performed using Analyst software (version 1.6.2, AB Sciex).

Table 1S

List of selected multiple reaction monitoring parameters, declustering potential (DP), collision energy (CE), and collision cell exit potential (CXP) for docetaxel and paclitaxel

| **Analyte** | **Mass** | **Precursor** | **Product ion** | **DP** | **CE** | **CXP** |
| --- | --- | --- | --- | --- | --- | --- |
| **Docetaxel** | **807.9** | **830.0** | **549.1^a^** | **183** | **35** | **21** |
|  |  |  | **304.0^b^** | **183** | **32** | **28** |
| **Paclitaxel** | **853.9** | **876.2** | **591.0^a^** | **230** | **34** | **22** |
|  |  |  | **308.0^b^** | **230** | **39** | **33** |

^a^ qualifier ion; ^b^ quantifier ion.

**(A)**

**(B)**

Figure 1S

Multiple reaction monitoring (MRM) chromatograms of blank plasma spiked with (A) docetaxel (10.8 ng/mL) and (B) the internal standard (IS; paclitaxel at 59.0 ng/mL). Respective MRM transitions for docetaxel and the IS were m/z 830.0 → 304.0 and 876.2 → 308.0.

Figure 2S

Chromatogram of the lower limit of quantification (LLOQ) for docetaxel in plasma.

Figure 3S

Plasma docetaxel concentration profiles of Tynen and docetaxel micelles in SD rats after an intravenous dose of 10 mg/kg. (*n*=3).

**(A)**

**SD rat plasma**

**↓ + docetaxel standard solutions**

**Mix 100 sec**

**↓**

**Incubate 1 hr, 37 °C**

**↓ + 1% formic acid**

**↓ + internal standard**

**Mix 60 sec**

**↓**

**Load onto Oasis HLB SPE**

**↓ Wash with 1% Formic acid**

**↓ Elute with methanol**

**Evaporate to dryness under N_2_**

**Reconstitute with 90% MeOH**

**↓**

**Inject onto LC/MS/MS**

**(Measure total docetaxel concentration, C_TOT_)**

**(B)**

**SD rat plasma**

**↓ + docetaxel standard solutions**

**Mix 100 sec**

**↓**

**Incubate 1 hr, 37 °C**

**↓ultrafiltration**

**Centrifuged 20 min (3270 x g, 4 °C)**

**↓**

**Supernatant**

**↓ + 1% formic acid**

**↓ + internal standard**

**Mix 60 sec**

**↓**

**Load onto Oasis HLB SPE**

**↓ Wash with 1% Formic acid**

**↓ Elute with methanol**

**Evaporate to dryness under N_2_**

**Reconstitute with 90% MeOH**

**↓**

**Inject onto LC/MS/MS**

**(Measure unbound docetaxel concentration, C_UF_)**

Figure 4S

Processing procedures of (A) unfiltered and (B) ultrafiltered plasma samples for determining the concentrations of total and unbound docetaxel in plasma.
